# Differential Diagnosis of Oral Salivary Gland Carcinoma and Squamous Cell Carcinoma Using Quantitative Dynamic Contrast-Enhanced MRI

**DOI:** 10.3390/jcm15020822

**Published:** 2026-01-20

**Authors:** Kunjie Zeng, Yanqin Zeng, Xinyin Chen, Siya Shi, Guoxiong Lu, Yusong Jiang, Xing Wu, Lingjie Yang, Zhaoqi Lai, Jiale Zeng, Yun Su

**Affiliations:** 1 Department of Radiology, Sun Yat-Sen Memorial Hospital, Sun Yat-Sen University, No. 107 Yanjiang Road West, Guangzhou 510120, China; zengkj6@mail.sysu.edu.cn (K.Z.); zengyq29@mail.sysu.edu.cn (Y.Z.); chenxy656@mail.sysu.edu.cn (X.C.); lugx7@mail.sysu.edu.cn (G.L.); jiangys5@mail.sysu.edu.cn (Y.J.); wuxing9@mail.sysu.edu.cn (X.W.); yanglj67@mail.sysu.edu.cn (L.Y.); 2 Department of Radiology, The First Affiliated Hospital, Sun Yat-Sen University, Guangzhou 510060, China; shisy@mail.sysu.edu.cn

**Keywords:** oral squamous cell carcinoma, minor salivary gland carcinomas, dynamic contrast-enhanced MRI, quantitative MRI

## Abstract

**Background/Objectives:** Preoperative differentiation between oral squamous cell carcinoma (SCC) and minor salivary gland carcinoma (SGC) remains clinically challenging due to overlapping imaging characteristics. This study aimed to develop a diagnostic model based on quantitative dynamic contrast-enhanced MRI (qDCE-MRI) parameters to distinguish SCC from SGC prior to surgery. **Methods:** Patients with histopathologic confirmed SCC or minor SGC who underwent preoperative 3.0T qDCE-MRI were recruited. Clinical characteristics and pharmacokinetic parameters, including volume transfer constant (K^trans^), reverse reflux rate constant (K_ep_), volume fraction of extravascular extracellular space (V_e_), plasma volume fraction (V_p_), time to peak (TTP), maximum concentration (MAXConc), maximal slope (MAXSlope), and area under the concentration-time curve (AUCt), along with the apparent diffusion coefficient (ADC), were extracted. Univariate and multivariable logistic regression analyses were performed to identify independent discriminators. Diagnostic performance was assessed using receiver operating characteristic analysis, and model comparisons were conducted with the DeLong test. Interobserver agreement was evaluated using intraclass correlation coefficients (ICC). **Results:** All qDCE-MRI parameters demonstrated excellent interobserver agreement (ICC range, 0.82–0.94). Multivariable analysis identified K_ep_ (OR = 2620.172, *p* = 0.001), maximal slope (OR = 1.715, *p* = 0.024), and tumor location (OR = 5.561, *p* = 0.027) as independent predictors. The qDCE-MRI model achieved superior diagnostic performance compared with the clinical model (AUC: 0.945 vs. 0.747; *p* = 0.012). **Conclusions:** A qDCE-MRI–based model incorporating K_ep_ and MAXSlope was shown to provide excellent accuracy for preoperative differentiation between oral SCC and minor SGC.

## 1. Introduction

Oral cancer is a common malignancy worldwide [1], with squamous cell carcinoma (SCC) being the most prevalent type, originating in the squamous cells of the oral mucosa. Salivary gland carcinomas (SGC), although less common, have gained significant attention due to their diverse subtypes and distinct tumor biology [2,3]. Surgical management for SCC typically involves ensuring a margin greater than 5 mm to reduce recurrence. While surgical management for SCC typically aims for clear margins greater than 5 mm to reduce recurrence [4], the required margin for SGC is more nuanced and depends on histological subtype and grade. For instance, low-grade mucoepidermoid carcinoma may be adequately treated with closer margins, whereas adenoid cystic carcinoma, known for perineural invasion, often necessitates wider resection [5,6]. Postoperative radiotherapy is commonly used for SCC with high-risk factors such as close/positive margins or lymph node involvement [7]. In SGC, its use is typically reserved for high-grade tumors, advanced T-stage, perineural invasion (particularly in adenoid cystic carcinoma), or positive surgical margins [8,9]. The five-year survival rate for SCC ranges from 40% to 63% [10], while SGC has a more favorable survival rate of 86% to 92% [4,11]. These differences highlight the need for accurate diagnosis and tailored treatment strategies for both cancers.

Current clinical guidelines recommend using fine-needle aspiration cytology (FNAC) or ultrasound-guided core needle biopsy (CNB) [12]. FNAC, while non-invasive, often has limited sensitivity (reported ranges from 77% to 86%) [13] and a high rate of non-diagnostic or inconclusive results (up to 15–20%) [14] in diagnosing salivary gland tumors [15]. In contrast, despite its diagnostic superiority [16], CNB is still not considered the reference standard due to its invasiveness, possible false negatives related to tumor heterogeneity [17], and concerns, though low-level and controversial, regarding tumor seeding [18]. Radiological imaging plays a crucial role in the diagnosis and staging of oral cancers. However, preoperative differentiation remains clinically challenging due to overlapping radiological features on conventional imaging modalities such as contrast-enhanced MRI. Both SCC and SGC can present as enhancing masses with infiltrative borders. In particular, infiltrative borders, a hallmark of SCC, can also be observed in low-grade SGCs such as adenoid cystic carcinoma [4]. Ultrasound provides real-time tumor assessment [19] but is operator-dependent and limited in evaluating deep tumors or areas like the floor of the mouth [20]. Therefore, utilizing more advanced imaging techniques to effectively differentiate SCC from SGC is crucial for clinical management. Quantitative dynamic contrast-enhanced magnetic resonance imaging (qDCE-MRI) is an advanced imaging technique that offers a comprehensive assessment of tumor vascularity and perfusion by providing a range of pharmacokinetic parameters. These parameters are fundamental in characterizing the microvascular environment of tumors, which is crucial for understanding their biological behavior [21]. Unlike conventional magnetic resonance imaging (MRI), which primarily provides morphological information, qDCE-MRI offers a functional assessment of tumor perfusion and microvascular permeability, which are critical in tumor differentiation. SCC is characterized by aggressive angiogenesis, often exhibiting higher K^trans^ and K_ep_ values due to increased vascular permeability [22,23]. In contrast, SGC, particularly adenoid cystic carcinoma, demonstrates lower perfusion rates with potentially distinct kinetic profiles [24,25,26]. Furthermore, qDCE-MRI is less susceptible to variability introduced by differences in MRI scanner hardware, scan sequences, and contrast agent protocols, making it a more reliable tool for clinical and research applications [21,27,28]. Previous research has demonstrated the high efficacy of qDCE-MRI in analyzing the aggressiveness of SGC [29]. The technique has also been successfully applied to identify small salivary gland tumors as benign or malignant [30], providing valuable preoperative information that can guide surgical planning and treatment strategies. Despite the established utility of qDCE-MRI in SGC, there is a relative scarcity of studies directly exploring its application in differentiating SCC from SGC. A few studies have employed DCE-MRI in head and neck cancers [21,31,32,33], but focused quantitative comparisons between oral SCC and minor SGC using pharmacokinetic parameters remain limited.

Therefore, we conducted a prospective study developing a qDCE-MRI-based diagnostic model for SCC and minor SGC differentiation. By integrating functional parameters, we aimed to provide radiologists and surgeons with a non-invasive tool to guide preoperative planning, potentially reducing diagnostic delays and unnecessary invasive procedures.

## 2. Methods

### 2.1. Patients

**Figure 1:**
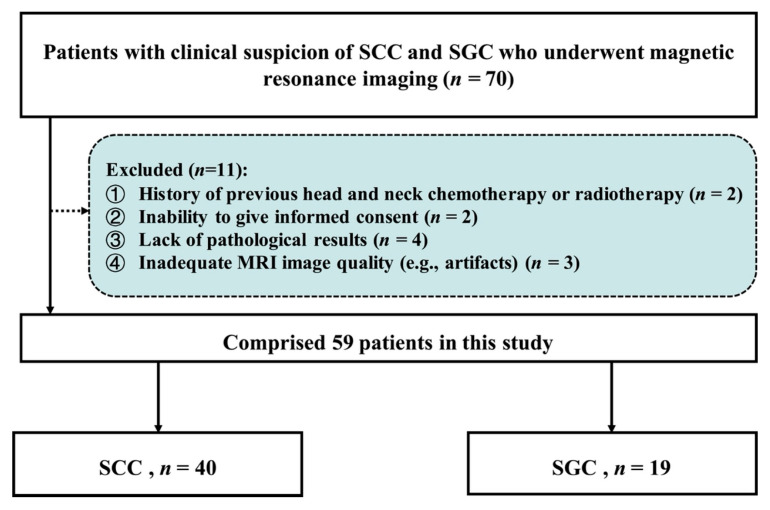
Process for inclusion of SGC and SCC patients based on pathological findings. SCC = squamous cell carcinoma; SGC = salivary gland carcinoma.

This prospective cohort study enrolled consecutive patients who were initially identified based on clinical suspicion (e.g., presence of a persistent oral ulcer, mass, or swelling) and/or radiologic suspicion on contrast-enhanced CT (e.g., a soft-tissue mass with irregular margins and heterogeneous enhancement in the oral cavity), between May 2023 and October 2024. The study protocol received ethical approval from the Institutional Review Board of Sun Yat-Sen Memorial Hospital, Sun Yat-Sen University (Guangzhou, China; Approval No. SYSKY-2023-432-02), with written informed consent obtained from all participants. Inclusion criteria required: (1) histopathological confirmation of SGC or SCC through postoperative specimen analysis, and (2) completion of preoperative qDCE-MRI within 7 days prior to surgical resection. Exclusion criteria included prior biopsy of the lesion, previous chemotherapy or radiotherapy in the head and neck region, lesions with a maximum diameter of less than 1 cm, significant motion artifacts on MRI, contraindications to gadolinium-based contrast agents or MRI (e.g., metal implants), and inability to provide informed consent. Figure 1 illustrates the flowchart for participant enrollment. Out of 70 participants recruited, 11 were excluded. Finally, 59 participants were included in the analysis. Clinical and pathological features were recorded, including patient sex, age, pathological type, differentiation grade, lymph node metastasis, tumor location, and tumor diameter (measured as the maximum diameter on axial T2-weighted MRI).

### 2.2. MR Imaging

All subjects underwent preoperative qDCE-MRI examinations using a 3.0 Tesla Achieva scanner (Philips Medical Systems, Amsterdam, The Netherlands) equipped with a 16-channel neurovascular coil. The imaging protocol, conducted within 7 days prior to surgical resection, comprised standard multiplanar sequences, diffusion-weighted imaging (DWI), and dynamic contrast-enhanced acquisitions. Anatomical imaging included axial and sagittal T1-weighted imaging (TR/TE = 628/18 ms) as well as axial T2-weighted sequences (TR/TE = 2643/90 ms), supplemented by coronal fat-suppressed T2-weighted imaging (TR/TE = 3000/60 ms), all performed with a 90° flip angle, 220 × 220 mm^2^ to 230 × 230 mm^2^ field of view (FOV), 5.0 mm slice thickness, and 1.0 mm interslice gap. DWI was performed using a single-shot spin-echo echo-planar imaging (SE-EPI) sequence with the following parameters: TR/TE = 8000/78 ms, field of view = 250 × 250 mm^2^, matrix = 128 × 128, slice thickness = 5.0 mm with 0.5 mm gap, and b-values of 0 and 1000 s/mm^2^. Apparent diffusion coefficient (ADC) maps were automatically generated and used for subsequent quantitative analysis. Dynamic contrast-enhanced imaging employed a 3D T1-weighted high-resolution isotropic volumetric excitation (THRIVE) sequence with TR/TE = 3.1/1.5 ms, 12° flip angle, 230 × 230 mm^2^ FOV, and 8.0 mm slice thickness (4.0 mm gap). The dynamic series encompassed 110 phases with 3 s temporal resolution, preceded by T1 mapping using variable flip angles (2°, 4°, 7°, 9°, 12°). Intravenous administration of 0.1 mmol/kg Gd-DTPA-BMA (Omniscan, GE Healthcare, Carrigtohill, Ireland) was performed via antecubital vein using a Spectris dual-head power injector (Medrad, Pittsburgh, PA, USA) at 3 mL/s, followed by 20 mL saline flush, with the entire dynamic acquisition completed within 5.5 min. Post-contrast conventional T1-weighted imaging in axial, sagittal, and coronal planes replicated pre-contrast scan parameters.

### 2.3. Imaging Processing

qDCE-MRI data processing was conducted using the Omni Kinetics quantitative analysis platform (GE Healthcare). To address motion artifacts, non-rigid registration with free-form deformation algorithms was applied for spatial alignment. Pharmacokinetic modeling was performed according to the extended Tofts two-compartment model [34]. with individualized arterial input functions (AIFs) obtained from the ipsilateral carotid arteries using variable flip angle acquisition. Two board-certified head and neck radiologists (4 and 10 years of subspecialty experience) independently performed whole-lesion segmentation through slice-wise manual delineation of volumetric regions of interest (ROIs). They ensured they remained blinded to the histopathological outcomes when defining three-dimensional volumes of interest (3D VOIs). The ROIs excluded large feeding vessels and areas of necrosis. The qDCE-MRI parameters that were included in the analysis were volume transfer constant (K^trans^), reverse reflux rate constant (K_ep_), volume fraction of extravascular extracellular space (V_e_), plasma volume fraction (V_p_), time to peak (TTP), maximum concentration (MAXConc), maximal slope (MAXSlope), and area under the concentration-time curve (AUCt).

### 2.4. Statistical Analysis

All analyses were performed using R software v3.6.0 (R Foundation, Austria). Continuous variables are expressed as mean ± standard deviation (SD). Normality was assessed with the Shapiro–Wilk test. Interobserver agreement for ADC values and qDCE-MRI parameters was evaluated using intraclass correlation coefficients (ICC), with consensus values from two radiologists used for subsequent analyses. Univariate logistic regression identified candidate discriminators, followed by multivariate analysis with stepwise variable selection established independent predictors and constructed the final model. Diagnostic performance was quantified by receiver operating characteristic (ROC) curve analysis, including the area under the curve (AUC), sensitivity, specificity, positive predictive value (PPV), negative predictive value (NPV), and accuracy. DeLong’s test compared AUC differences between clinical information and MRI parameter models.

## 3. Results

### 3.1. Study Population

A total of 59 patients (36 males, 23 females; age range 29–75 years) were enrolled, comprising 19 with SGC (10 males, mean age 48 ± 11 years) and 40 with SCC (26 males, mean age 56 ± 13 years). SCC patients were significantly older than SGC patients (*p* = 0.025) and exhibited higher lymph node metastasis (*p* = 0.018, Table 1). Tumor location differed significantly: SCC predominated in the tongue/buccal regions, whereas SGC occurred more frequently in the jaw (*p* = 0.004). No intergroup differences were observed in sex, differentiation grade, and tumor diameter (all *p* > 0.05). ICC analysis demonstrated excellent interobserver agreement for both pharmacokinetic parameters and ADC values (ICC range 0.82–0.94). All pharmacokinetic parameters—including K^trans^, K_ep_, MAXConc, MAXSlope, TTP, V_e_, V_p_, and AUCt—demonstrated significant differences between SCC and SGC (all *p* < 0.05), while ADC values showed no intergroup difference (*p* > 0.05). No intergroup differences were observed in sex, differentiation grade, and MRI-measured tumor diameter (all * *p* * > 0.05), suggesting that size alone is not a distinguishing factor.

### 3.2. Univariate and Multivariate Regression Analyses

**Figure 2:**
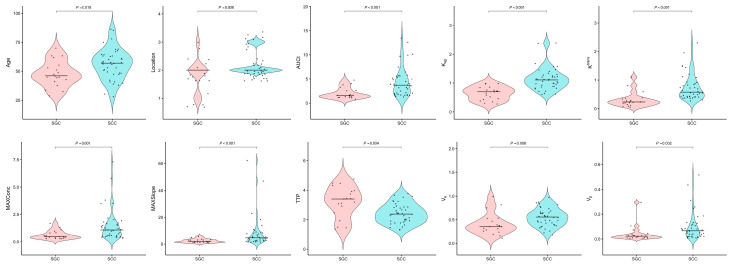
The box plot shows the clinical characteristics and qDCE-MRI parameters of SGC and SCC. AUCt = area under the concentration-time curve; K_ep_ = reflux rate; K^trans^ = volume transfer constant; MAXConc = maximum concentration; MAXSlope = maximal slope; TTP = time to peak; V_e_ = volume fraction of the extravascular extracellular matrix; V_p_ = volume fraction of plasma.

**Figure 3:**
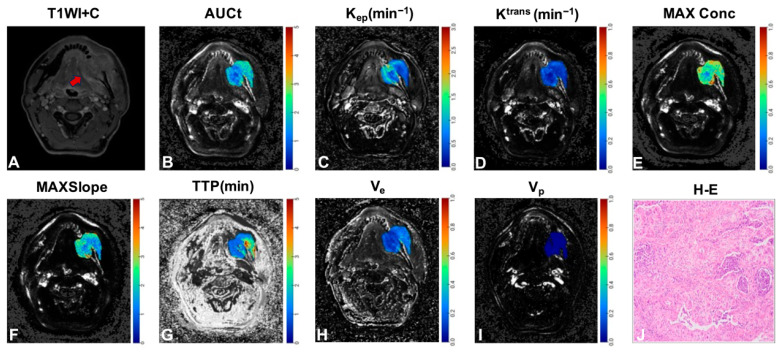
A case of gingival carcinoma. (**A**) Axial T1-weighted enhanced image of squamous cell carcinoma. (**B**–**I**) Corresponding color-encoded overlay maps of AUCt, K_ep_, K^trans^, MaxConc, MaxSlope, TTP, V_e_, and V_p_ are shown, respectively. (**J**) Representative photomicrographs of H-E-stained sections at 200× magnification are shown. The averages of AUCt, K_ep_, K^trans^, MaxConc, MaxSlope, TTP, V_e_, and V_p_ measured by the two readers were 3.189, 1.205 min^−1^, 0.596 min^−1^, 0.846, 5.702, 1.649 min, 0.179, and 0.015, respectively. AUCt = area under the concentration-time curve; K_ep_ = reflux rate; K^trans^ = volume transfer constant; MAXConc = maximum concentration; MAXSlope = maximal slope; TTP = time to peak; V_e_ = volume fraction of the extravascular extracellular matrix; V_p_ = volume fraction of plasma; H-E = hematoxylin and eosin staining.

**Figure 4:**
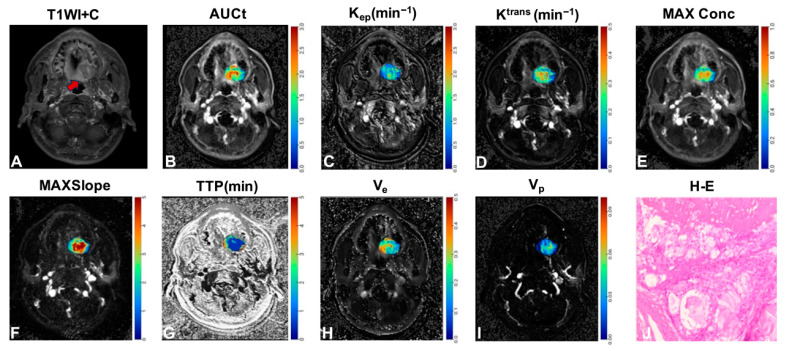
A case of moderate malignant mucoepidermoid carcinoma. (**A**) Axial T1-weighted enhanced image of salivary gland carcinoma. (**B**–**I**) Corresponding color-encoded overlay maps of AUCt, K_ep_, K^trans^, MaxConc, MaxSlope, TTP, V_e_, and V_p_ are shown, respectively. (**J**) Representative photomicrographs of H-E-stained sections at 200× magnification are shown. The averages of AUCt, K_ep_, K^trans^, MaxConc, MaxSlope, TTP, V_e_, and V_p_ measured by the two readers were 1.616, 0.967 min^−1^, 0.199 min^−1^, 0.475, 3.011, 1.500 min, 0.240, and 0.026, respectively. AUCt = area under the concentration-time curve; K_ep_ = reflux rate; K^trans^ = volume transfer constant; MAXConc = maximum concentration; MAXSlope = maximal slope; TTP = time to peak; V_e_ = volume fraction of the extravascular extracellular matrix; V_p_ = volume fraction of plasma. H-E = hematoxylin and eosin staining.

Univariate logistic regression identified both age (*p* = 0.031) and tumor location (*p* = 0.014) as significant discriminators between SCC and SGC (Table 2, Figure 2). Among the qDCE-MRI parameters, K^trans^ (*p* = 0.002), K_ep_ (*p* < 0.001), AUCt (*p* = 0.006), MAXConc (*p* = 0.014), MAXSlope (*p* = 0.005), TTP (*p* = 0.003), and V_e_ (*p* = 0.023) also showed significant discriminatory ability (Table 3, Figure 2). In multivariate analysis, tumor location remained the sole independent clinical predictor (*p* = 0.014; Table 2), while the qDCE-MRI parameters revealed MAXSlope (*p* = 0.024) and K_ep_ (*p* = 0.001) as independent imaging biomarkers (Table 3). Figure 3 and Figure 4 present color-encoded overlay maps of pharmacokinetic parameters in representative SCC and SGC cases, respectively. In the SCC case (Figure 3), the K_ep_ value (1.205 min^−1^) is significantly higher than that in the SGC case (0.967 min^−1^; Figure 4). A similar difference is observed in the MAXSlope values (5.702 vs. 3.011).

### 3.3. Model Performance and Comparison

**Figure 5:**
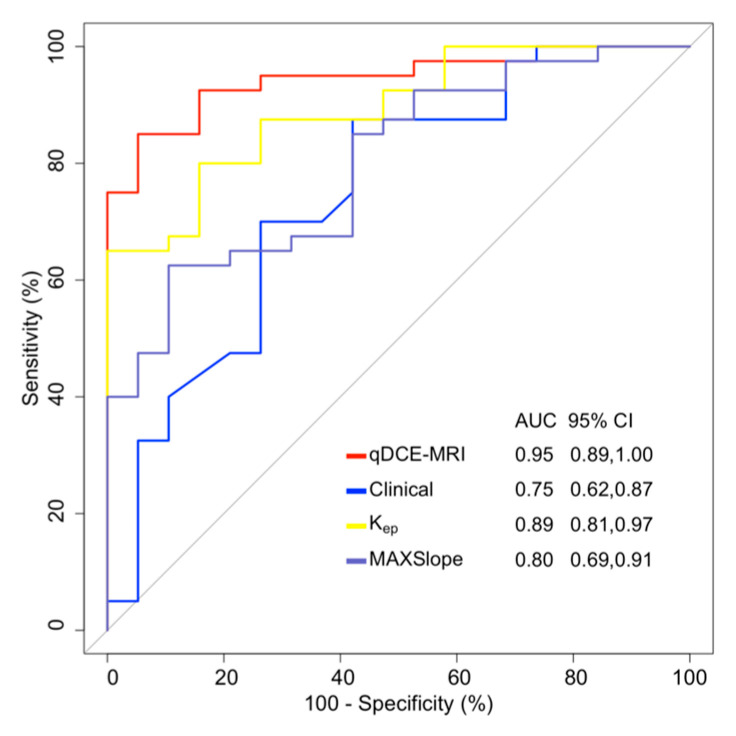
ROCs of single qDCE-MRI parameter model, clinical model, and qDCE-MRI model for distinguishing SCC and SGC. AUC = area under the ROC curve; CI = confidence interval; K_ep_ = reflux rate; MAXSlope = maximal slope; qDCE-MRI = quantitative dynamic contrast-enhanced magnetic resonance imaging.

Based on the aforementioned independent predictors, we constructed four distinct models: a clinical model (incorporating tumor location), a qDCE-MRI multiparametric model (integrating K_ep_ and MAXSlope), along with two single-parameter models (K_ep_ and MAXSlope, respectively). As shown in Table 4 and Figure 5, the clinical model demonstrated an AUC of 0.747 (95% CI: 0.622–0.873), with an accuracy of 78%, sensitivity of 88%, and specificity of 58%. The qDCE-MRI model achieved superior diagnostic performance, exhibiting an AUC of 0.945 (95% CI: 0.889–1.000), accuracy of 88%, sensitivity of 85%, and specificity of 95%. For the K_ep_ model, the AUC was 0.891 (95% CI: 0.810–0.972), with an accuracy of 76%, sensitivity of 65%, and specificity of 100%. Similarly, the MAXSlope model yielded an AUC of 0.800 (95% CI: 0.688–0.912), accuracy of 71%, sensitivity of 63%, and specificity of 89%.

The DeLong test revealed that the qDCE-MRI model demonstrated a significantly higher AUC than the clinical model (0.945 vs. 0.747; *p* = 0.012). Compared to the K_ep_ model, it showed comparable AUC performance (0.945 vs. 0.891; *p* = 0.086), while significantly outperforming the MAXSlope model (0.945 vs. 0.800; *p* = 0.004).

## 4. Discussion

This study suggests that qDCE-MRI holds promise in distinguishing between minor SGC and SCC, highlighting its potential as a valuable tool in oral oncology. Significant differences in pharmacokinetic parameters between minor SGC and SCC were identified, with K_ep_ and MAXSlope emerging as independent predictors for distinguishing the two. Their integration further improves diagnostic performance.

Minor SGC refers to malignant tumors originating from the numerous small salivary glands widely distributed within the oral submucosa (e.g., the palate and related subregions), while SCC arises from the squamous epithelium of the oral mucosa. However, preoperative differentiation remains clinically challenging [35]. First, both tumors may present as submucosal masses with unclear morphological features on contrast-enhanced MRI, with the lesion borders potentially being ill-defined, especially when stromal infiltration or peritumoral inflammation is present [36]. Second, enhancement patterns often overlap: SCC frequently shows heterogeneous enhancement due to necrosis and uneven vascular distribution, while minor salivary gland carcinomas may also display similar heterogeneous enhancement due to the mixture of solid and stromal components [37]. Third, certain subtypes of minor SGC, particularly adenoid cystic carcinoma, may exhibit infiltrative growth and the potential for perineural or submucosal spread, leading to infiltrative margins on conventional imaging that mimic those of SCC [38]. Finally, both tumors may show overlapping signal intensities on T1- and T2-weighted sequences, where SCC typically presents with low T1 signal, slightly elevated T2 signal, and variable enhancement, while minor SGCs show near-equal T1 signal, mildly elevated T2 signal, and significant enhancement. This overlap limits the diagnostic value of morphological evaluation alone [39]. Collectively, these overlapping features highlight the necessity for quantitative functional imaging, such as qDCE-MRI.

In the present study, advanced acquisition strategies reported in the previous literature were adopted in the design of the qDCE-MRI protocol to enhance the reliability and reproducibility of pharmacokinetic parameter quantification [21,27,40,41]. Specifically, during baseline T1 mapping, a multiple flip-angle acquisition scheme using five variable flip angles was applied, which effectively corrects the nonlinear relationship between MRI signal intensity and contrast agent concentration, thereby providing more accurate and robust T1 mapping and pharmacokinetic parameter estimation within a relatively short acquisition time [19,21,27]. The temporal resolution of 3 s used in this study represents an optimal balance between spatial resolution and signal quality, facilitating improved image clarity while accurately capturing the hemodynamic behavior of the contrast agent [40]. In addition, compared with population-based AIFs, the use of individually derived AIFs in this study more accurately reflects patient-specific arterial input characteristics, further improving the accuracy of quantitative analysis [40].

The significant age difference observed between SCC and SGC patients aligns with previous reports [4], indicating that SCC typically appears at an older age, whereas SGC tends to occur in slightly younger populations. SCC patients were also more likely to have lymph node involvement, which is primarily due to SCC’s higher local invasiveness. SCC mainly occurs in the oral cavity, where its anatomical location makes it more prone to early spread through the lymphatic system. In contrast, SGC originates in the salivary glands, initially having less exposure to the lymphatic system, resulting in a relatively lower incidence of lymph node metastasis [42,43]. Additionally, tumor location also showed discriminatory value between minor SGC and SCC, likely reflecting differences in tissue of origin. Specifically, minor SGC arises from salivary gland tissue, which is more abundant in regions such as the hard palate and posterior tongue, whereas SCC originates from the oral mucosal epithelium, which is widely distributed throughout the oral cavity (e.g., the tongue, floor of the mouth, buccal mucosa, hard and soft palate, gingiva, and lips) [4].

In our study, qDCE-MRI parameters, including K^trans^, K_ep_, AUCt, MAXConc, MAXSlope, TTP, V_e_, and V_p_, demonstrated significant discriminative power in differentiating SCC from SGC. Firstly, the significant difference in AUCt indicates that the cumulative contrast agent concentration in SCC patients is significantly higher than in SGC patients, possibly due to the higher angiogenesis and blood flow in SCC tumors. The differences in K_ep_ and K^trans^ suggest that SCC tumors have higher contrast agent transfer rates and vascular permeability than SGC, reflecting the higher angiogenesis and permeability characteristics of SCC tumors [44]. The significant differences in MAXConc and MAXSlope further support the conclusion of higher angiogenic activity in SCC tumors. MAXConc reflects the peak contrast agent concentration within the tumor, while MAXSlope reflects the rate at which the contrast agent concentration reaches its peak, both of which are significantly higher in SCC patients than in SGC patients [45]. The TTP results show that SGC patients have a longer TTP, indicating that the contrast agent takes longer to reach its peak concentration within SGC tumors, consistent with the lower angiogenesis and slower blood flow in SGC [27]. The differences in V_e_ and V_p_ also reveal that SCC tumors have more complex and extensive vascular structures and volumes, possibly due to their higher invasiveness and angiogenic capabilities [46]. Interestingly, while all analyzed qDCE-MRI parameters showed significant differences, ADC values did not. This insensitivity may be attributed to overlapping tissue microstructures: both SCC and SGC can contain areas of high cellularity, necrosis, or stromal components (e.g., mucin or collagen) that influence water diffusion in complex ways, leading to similar mean ADC values [47]. Furthermore, the region-of-interest analysis might average out microscopic histological differences that perfusion parameters like K_ep_ and MAXSlope are more sensitive to. It is important to note that minor SGC subtypes (e.g., adenoid cystic carcinoma vs. mucoepidermoid carcinoma) are heterogeneous, and their perfusion characteristics may vary, a factor not analyzed in the current study due to sample size constraints [48].

In our study, multivariate analysis revealed that tumor location was the only independent predictor for distinguishing SCC from SGC, indicating that it contains a wealth of discriminative information that may overlap with other clinical features, such as age and lymph node involvement, potentially masking the effects of other variables in multivariable adjustments. Additionally, qDCE-MRI parameters, MAXSlope and K_ep_, emerged as independent discriminators. Although other qDCE-MRI parameters were also significant in univariate analysis, MAXSlope and K_ep_ provided sufficient information in multivariate analysis, rendering the contributions of other parameters insignificant. These findings suggest that MAXSlope and K_ep_ capture key hemodynamic characteristics related to tumor type and remain statistically independent in multivariable analysis. Histopathologic angiogenesis markers most commonly include microvessel density (MVD) quantified by endothelial immunohistochemistry (e.g., CD31/CD34) and VEGF-related signaling. These markers directly measure angiogenic activity and remain important tissue-based indicators in head and neck squamous cell carcinoma research [49,50]. In contrast, MAXSlope and K_ep_ provide whole-tumor functional assessments of contrast agent inflow kinetics and reflux/permeability-related behavior, which are biologically influenced by microvessel density, vessel maturity, and endothelial permeability [51,52]. The Delong test results showed a significant difference between the qDCE-MRI and clinical models (AUC: 0.945 vs. 0.747; *p* = 0.012), confirming the superior discriminative ability of the qDCE-MRI parameters. This indicates that qDCE-MRI provides a more refined and detailed tumor characterization that traditional clinical predictors might not capture. While clinical predictors like location offer anatomical clues, qDCE-MRI parameters like K_ep_ and MAXSlope directly quantify physiological processes (angiogenesis, vascular permeability) that are central to tumor biology and may not be inferable from anatomy alone [53]. The combination of MAXSlope and K_ep_ (qDCE-MRI model) provided a higher (though not statistically significant, *p* = 0.086) AUC compared to K_ep_ alone (0.945 vs. 0.891) and a significantly higher AUC than MAXSlope alone (*p* = 0.004), suggesting complementary information from these two hemodynamic parameters. The high accuracy and specificity of the qDCE-MRI model highlight its potential in improving personalized diagnostic and treatment strategies for cancer patients. For example, more accurate differentiation between SCC and minor SGC could influence decisions regarding surgical margin planning, the extent of neck dissection, and the consideration of adjuvant radiotherapy [54,55,56,57].

Previous studies have explored the application of qDCE-MRI parameters in head and neck tumors, but most have focused on tumor characterization, staging, or treatment monitoring, rather than specifically distinguishing oral SCC from minor SGC [29,58,59]. For instance, Chikui et al. studied a cohort of 85 oral SCC patients and found that DCE-MRI pharmacokinetic parameters such as K^trans^ and v_e_ were correlated with clinical N stage, suggesting that perfusion characteristics are linked to tumor invasiveness [58]. However, the study did not evaluate its diagnostic value for differentiating SCC from other tumor types. Similarly, Guo et al. confirmed that qDCE-MRI parameters, including K^trans^ and K_ep_, could serve as biomarkers to predict the pathological stage of tongue SCC [29]. While this study reinforced the value of perfusion imaging for assessing tumor biology, it focused only on SCC staging and did not compare it with other malignancies. Hisatomi et al. reported that conventional DCE-MRI, using contrast index curves such as T_max_ and washout rate (WR), could differentiate benign from malignant major salivary gland tumors [59]. Unlike prior studies that primarily examined perfusion metrics within a single tumor type, our findings suggest that qDCE-MRI may help differentiate minor SGC from oral SCC in a mixed clinical cohort. This observation adds supportive evidence for the potential subtype-specific utility of qDCE-MRI in the preoperative evaluation of these two oral malignancies.

Several limitations of this study should be acknowledged. Firstly, the relatively modest sample size and single-center design may limit the generalizability of our findings, and external validation in independent, multicenter cohorts is required. Secondly, although significant differences in qDCE-MRI parameters were observed between SCC and minor SGC, subgroup analyses within minor SGC were not performed. Given the biological heterogeneity among minor SGC subtypes (e.g., adenoid cystic carcinoma and mucoepidermoid carcinoma), perfusion characteristics may vary across histologies, which warrants further investigation in larger datasets. Thirdly, variations in MRI acquisition protocols, scanner hardware, and post-processing approaches may influence quantitative parameter measurements. Therefore, future studies should emphasize protocol standardization, inter-scanner reproducibility testing, and assessment of interobserver variability to facilitate broader clinical applicability. Fourthly, whole-lesion segmentation was performed manually, which is time-consuming and may introduce operator dependence despite the use of experienced readers and blinding. Future work should evaluate semi-automated or fully automated segmentation methods to improve efficiency and reproducibility. Fifthly, the high diagnostic performance (AUC 0.945) of our model was derived from the same cohort used for feature selection and model building, without internal cross-validation or an independent external validation set. This can lead to optimistic performance estimates. External validation in a separate patient population is essential to confirm the model’s generalizability and clinical utility. Finally, this study focused on diagnostic performance and did not evaluate the impact of qDCE-MRI–based differentiation on clinical decision-making or patient outcomes. Prospective decision-impact and outcome-based studies are needed to determine the true clinical utility of incorporating qDCE-MRI into multidisciplinary management.

## 5. Conclusions

In conclusion, our study suggests that a qDCE-MRI diagnostic model incorporating MAXSlope and K_ep_ shows promising diagnostic accuracy for differentiating between SCC and minor SGC in this initial cohort. However, further validation in independent, multicenter populations is required to confirm generalizability. With standardized acquisition/analysis protocols and assessment of interobserver reproducibility, qDCE-MRI may serve as a useful adjunct to routine imaging. The potential clinical impact on treatment planning remains to be established, and future prospective studies should evaluate decision impact and patient outcomes when incorporating the model into multidisciplinary management.

## Figures and Tables

**Table 1:** Comparison of basic information between SCC and SGC.

Variables	SGC (*n* = 19)	SCC (*n* = 40)	*p*
Sex, *n* (%)			0.532
Male	10 (53)	26 (65)	
Female	9 (47)	14 (35)	
Age, Mean ± SD	47.89 ± 11.15	56.02 ± 13.32	0.018
Pathological type, *n* (%)			
Mucoepidermoid carcinoma	7 (37)	0 (0)	
Adenoid cystic carcinoma	10 (53)	0 (0)	
Salivary duct carcinoma	1 (5)	0 (0)	
Basal cell adenocarcinoma	1 (5)	0 (0)	
Squamous cell carcinoma	0 (0)	40 (100)	
Differentiation grade, *n* (%)			0.650
Well	5 (26)	12 (30)	
Moderate	11 (58)	25 (62)	
Poor	3 (16)	3 (8)	
Lymph node metastasis, *n* (%)			0.018
No	11 (58)	35 (88)	
Yes	8 (42)	5 (12)	
Tumor location, *n* (%)			0.004
Palatine	5 (26)	0 (0)	
Tongue	12 (63)	30 (75)	
Cheek/Gingiva	2 (11)	10 (25)	
Tumor diameter, Median (Q1, Q3)	24 (21.5, 49)	29 (21.75, 35.5)	0.615
ADC, Median (Q1, Q3)	1.21 (1, 1.3)	1.13 (1, 1.24)	0.615
AUCt, Median (Q1, Q3)	1.6 (1.24, 2.57)	3.73 (1.91, 5.57)	<0.001
K_ep_, Median (Q1, Q3)	0.69 (0.44, 0.79)	1.11 (0.88, 1.27)	<0.001
K^trans^, Median (Q1, Q3)	0.23 (0.17, 0.35)	0.58 (0.41, 0.89)	<0.001
MAXConc, Median (Q1, Q3)	0.47 (0.35, 0.82)	1.09 (0.54, 1.67)	0.001
MAXSlope, Median (Q1, Q3)	2.05 (1.43, 3.18)	4.76 (2.64, 8.37)	<0.001
TTP, Mean ± SD	3.21 ± 1.05	2.42 ± 0.67	0.004
V_e_, Mean ± SD	0.41 ± 0.23	0.55 ± 0.19	0.006
V_p_, Median (Q1, Q3)	0.02 (0.01, 0.03)	0.07 (0.04, 0.13)	0.002

Note: AUCt = area under the concentration-time curve; K_ep_ = reflux rate; K^trans^ = volume transfer constant; MAXConc = maximum concentration; MAXSlope = maximal slope; TTP = time to peak; V_e_ = volume fraction of the extravascular extracellular matrix; V_p_ = volume fraction of plasma. Tumor diameter is the maximum diameter measured on preoperative axial T2-weighted MRI.

**Table 2:** Univariate and multivariate logistic regression analysis of various clinical indicators to distinguish SCC and SGC.

	Univariable Logistic Regression				Multivariable Logistic Regression			
	*β* Value	OR	95% CI	*p*-Value	*β* Value	OR	95% CI	*p*-Value
Sex	−0.514	0.598	0.197, 1.816	0.365	-	-	-	-
Age	0.054	1.055	1.005, 1.108	0.031 *	0.049	1.050	0.995, 1.109	0.078
Differentiation grade	−0.339	0.713	0.282, 1.797	0.473	-	-	-	-
Tumor location	1.890	6.621	1.459, 30.040	0.014 *	1.716	5.561	1.218, 25.388	0.027 *
Tumor diameter	−0.025	0.976	0.938, 1.015	0.221	-	-	-	-
ADC	−0.916	0.400	0.055, 2.932	0.367	-	-	-	-

Note. CI = confidence intervals. * Indicates statistical significance; *p*-value < 0.05. SCC = squamous cell carcinoma; SGC = salivary gland carcinoma; ADC = apparent diffusion coefficient.

**Table 3:** Univariate and multivariate logistic regression analysis of various qDCE-MRI indicators to distinguish SCC and SGC.

	Univariable Logistic Regression				Multivariable Logistic Regression			
	*β* Value	OR	95% CI	*p*-Value	*β* Value	OR	95% CI	*p*-Value
AUCt	0.693	1.999	1.223, 3.266	0.006 *	-	-	-	-
K_ep_	7.107	1220.387	26.736, 55,706.271	<0.001 *	7.871	2620.172	20.890, 328,517.888	0.001 *
K^trans^	5.704	300.056	8.096, 11,120.452	0.002 *	-	-	-	-
MAXConc	1.772	5.885	1.434, 24.151	0.014 *	-	-	-	-
MAXSlope	0.577	1.780	1.185, 2.674	0.005 *	0.540	1.715	1.073, 2.741	0.024 *
TTP	−1.140	0.320	0.150, 0.681	0.003 *	-	-	-	-
V_e_	3.672	39.316	1.668, 926.882	0.023 *	-	-	-	-
V_p_	11.363	83,745.35	0.547, >999999	0.063	-	-	-	-

Note. CI = confidence intervals. * Indicates statistical significance; *p*-value < 0.05. SCC = squamous cell carcinoma; SGC = salivary gland carcinoma; AUCt = area under the concentration-time curve; K_ep_ = reflux rate; K^trans^ = volume transfer constant; MAXConc = maximum concentration; MAXSlope = maximal slope; TTP = time to peak; V_e_ = volume fraction of the extravascular extracellular matrix; V_p_ = volume fraction of plasma.

**Table 4:** Diagnostic performance of qDCE-MRI quantitative parameters and clinical features in distinguishing SCC and SGC.

Model	AUC [95% CI]	Sensitivity (%)	Specificity (%)	Accuracy (%)	PPV (%)	NPV (%)
qDCE-MRI	0.95 [0.89, 1.00]	85	95	88	97	75
Clinical	0.75 [0.62, 0.87]	88	58	78	81	69
K_ep_	0.89 [0.81, 0.97]	65	100	76	100	58
MAXSlope	0.80 [0.69, 0.91]	63	89	71	93	53

Note: AUC = area under the curve; CI = confidence intervals; NPV = negative predictive value; PPV = positive predictive value; SCC = squamous cell carcinoma; SGC = salivary gland carcinoma; qDCE-MRI = quantitative dynamic contrast-enhanced magnetic resonance imaging; K_ep_ = reflux rate; MAXSlope = maximal slope.

## Data Availability

The datasets generated and/or analyzed during the current study are available from the corresponding author on reasonable request.

## Abbreviations

SCC	Squamous cell carcinoma
SGC	Salivary gland carcinoma
qDCE-MRI	Quantitative dynamic contrast-enhanced MRI
K^trans^	Volume transfer constant
K_ep_	Reverse reflux rate constant
V_e_	Volume fraction of extravascular extracellular space
V_p_	Plasma volume fraction
TTP	Time to peak
MAXConc	Maximum concentration
MAXSlope	Maximal slope
AUCt	Area under the concentration-time curve
ADC	Apparent diffusion coefficient
AUC	Area under the ROC curve
ICC	Intraclass correlation coefficient
FNAC	Fine-needle aspiration cytology
CNB	Ultrasound-guided core needle biopsy
MRI	Magnetic resonance imaging
DWI	Diffusion-weighted imaging
THRIVE	T1-weighted high-resolution isotropic volumetric excitation
AIFs	Arterial input functions
ROIs	Volumetric regions of interest
3D VOIs	Three-dimensional volumes of interest
SD	Standard deviation
ROC	Receiver operating characteristic
PPV	Positive predictive value
NPV	Negative predictive value
